# Family-Based Association Analysis Confirms the Role of the Chromosome 9q21.32 Locus in the Susceptibility of Diabetic Nephropathy

**DOI:** 10.1371/journal.pone.0060301

**Published:** 2013-03-29

**Authors:** Marcus G. Pezzolesi, Jackson Jeong, Adam M. Smiles, Jan Skupien, Josyf C. Mychaleckyj, Stephen S. Rich, James H. Warram, Andrzej S. Krolewski

**Affiliations:** 1 Research Division, Joslin Diabetes Center, Boston, Massachusetts, United States of America; 2 Department of Medicine, Harvard Medical School, Boston, Massachusetts, United States of America; 3 Center for Public Health Genomics, University of Virginia School of Medicine, Charlottesville, Virginia, United States of America; RIKEN Center for Genomic Medicine, Japan

## Abstract

A genome-wide association scan of type 1 diabetic patients from the GoKinD collections previously identified four novel diabetic nephropathy susceptibility loci that have subsequently been shown to be associated with diabetic nephropathy in unrelated patients with type 2 diabetes. To expand these findings, we examined whether single nucleotide polymorphisms (SNPs) at these susceptibility loci were associated with diabetic nephropathy in patients from the Joslin Study of Genetics of Nephropathy in Type 2 Diabetes Family Collection. Six SNPs across the four loci identified in the GoKinD collections and 7 haplotype tagging SNPs, were genotyped in 66 extended families of European ancestry. Pedigrees from this collection contained an average of 18.5 members, including 2 to 14 members with type 2 diabetes. Among diabetic family members, the 9q21.32 locus approached statistical significance with advanced diabetic nephropathy (*P* = 0.037 [adjusted *P* = 0.222]). When we expanded our definition of diabetic nephropathy to include individuals with high microalbuminuria, the strength of this association improved significantly (*P* = 1.42×10^−3^ [adjusted *P* = 0.009]). This same locus also trended toward statistical significance with variation in urinary albumin excretion in family members with type 2 diabetes (*P* = 0.032 [adjusted *P* = 0.192]) and in analyses expanded to include all relatives (*P* = 0.019 [adjusted *P* = 0.114]). These data increase support that SNPs identified in the GoKinD collections on chromosome 9q21.32 are true diabetic nephropathy susceptibility loci.

## Introduction

Increased urinary albumin excretion, in both the microalbuminuric and proteinuric ranges, is a hallmark of diabetic nephropathy (DN) [1], [2]. Clinically, DN is a progressive disease that advances through characteristic stages. For many diabetic patients, elevated urinary albumin excretion is coupled with declining renal function. In a large proportion of these individuals, renal function continues to deteriorate until end-stage renal disease (ESRD) is reached.

Despite a large body of evidence that favors a genetic basis for susceptibility to DN, identification of the genetic factors that contribute to its risk has proven challenging [3]–[12]. While no single, major DN susceptibility gene has yet been identified, growing support for several loci identified though genome-wide surveys of common genetic variants has recently begun to emerge [13]–[22]. Included among the studies contributing to this success is our recent genome-wide association (GWA) scan of unrelated type 1 diabetic (T1D) subjects from the Genetics of Kidneys in Diabetes (GoKinD) study collections [18]. In this report, we identified strong associations at several common single nucleotide polymorphisms (SNPs, minor allele frequencies >5%) located across four distinct chromosomal regions. Three of these loci, located on chromosome 9q21.32 near the *FRMD3* gene, chromosome 11p15.4 at the *CARS* gene, and chromosome 13q33.3 at the *MYO16*/*IRS2* locus, have since been confirmed in multiple diverse collections of unrelated T1D or type 2 diabetic (T2D) patients [18], [20], [22]. A more recent meta-analysis of T1D nephropathy, defined as end-stage renal disease (ESRD), in European-derived populations, however, failed to confirm these, as well as several other, previously reported genetic associations; reinforcing the need for further investigation of these and other loci to truly understand their role in the genetic basis of DN [23].

To address this need, we chose to extend our focused evaluation of the loci identified in GoKinD to a family-based association study of patients from the Joslin Study of Genetics of Nephropathy in Type 2 Diabetes Family Collection. In addition to dichotomized comparisons of DN status, we investigated whether any of these loci were associated with quantitative variation in urinary albumin in this collection.

## Materials and Methods

### Study Patients and Ethics Statement

The present study investigated 1,221 individuals (798 with direct genotype and phenotype information) from 66 extended families of European ancestry from the Joslin Study of Genetics of Nephropathy in Type 2 Diabetes Family Collection. The protocols and informed consent procedures used in this study were approved by the Committee on Human Subjects of the Joslin Diabetes Center.

The recruitment of the Joslin Study of Genetics of Nephropathy in Type 2 Diabetes Family Collection has previously been described [9], [11], [12], [24]. Briefly, between 1993 and 2003, families with an apparent autosomal dominant mode of inheritance of T2D, irrespective of their nephropathy status, were recruited to the Joslin Study of Genetics of Nephropathy in Type 2 Diabetes Family Collection through T2D probands receiving medical care at the Joslin Clinic. After obtaining informed written consent, trained recruiters administered previously described study protocols that included a structured interview, seated blood pressure measurements, and the collections of blood and urine samples. ESRD status for members of this collection was updated as of August 2008 through the United States Renal Data System.

### Classification of Nephropathy Status

Methods for measuring albumin and creatinine in a random urine sample for determination of the albumin-to-creatinine ratio (ACR) and defining normoalbuminuria, microalbuminuria, or proteinuria were described previously [25]. ACR values were used to assign albuminuria status to all individuals included in our analysis; individuals with ACR values less than 30 µg/mg, between 30 µg/mg and 300 µg/mg, between 100 µg/mg and 300 µg/mg, and above 300 µg/mg were considered normoalbuminuric, microalbuminuric, high microalbuminuric, and proteinuric, respectively. Individuals with ESRD were assigned ACR values of 3500 µg/mg. For quantitative trait analyses, a log transformation was applied to the measured/assigned ACR values.

### Genotyping

Six SNPs across the four loci identified in the GoKinD collections were selected for inclusion in the present study; including rs39075 on chromosome 7p14.3, rs1888747 and rs10868025 on chromosome 9q21.32, rs451041 on chromosome 11p15.4, and rs1411766 and rs9521445 on chromosome 13q33.3. Seven additional haplotype tagging SNPs (two on chromosomes 7p14.3, 11p15.4, and 13q33.3 and one on chromosome 9q21.32) were selected using Haploview [26] to capture the major haplotypes (haplotype frequencies ≥0.05) for the linkage disequilibrium (LD) blocks containing the SNPs identified in GoKinD. All thirteen SNPs were genotyped using Taqman (Applied Biosystems, Foster City, CA) technology by the Genetics Core of the Diabetes and Endocrinology Research Center at the Joslin Diabetes Center in accordance with the manufacturer's protocols.

### Statistical Analysis

Each SNP was tested for deviation from Hardy-Weinberg equilibrium using a chi-square goodness-of-fit test. Family-based single-marker association tests were performed using the FBAT software under an additive model using a conservative empirical variance estimator to test the null hypothesis of no linkage and no association [27]. For all dichotomous trait analyses, allele transmissions from parent to affected and unaffected family members were contrasted by weighting their contribution to the FBAT test statistic using the estimated population prevalence of DN among diabetic individuals (i.e., 30%; ‘Affected and Unaffected’ analyses). Family-based association testing of allele transmission from parents to only affected offsprings was also performed (i.e., ‘Affecteds Only’ analyses). The HBAT procedure in FBAT was used to estimate haplotype frequencies and perform haplotype-specific and global tests of association. *P*-values <8.33×10^−3^ (0.05/6) were considered statistically significant.

## Results

A total of 1,221 individuals from 66 extended families of European ancestry from the Joslin Study of Genetics of Nephropathy in Type 2 Diabetes Family Collection, including 798 (382 non-diabetic and 416 diabetic) members with direct genotype and phenotype information, were included in the present study. Pedigrees from these families included an average of 18.5 members, ranging in size from 6 to 39 members, and formed a total of 318 nuclear families. Four to 26 individuals from each family, including 2 to 14 members with diabetes, had DNA available for genotyping. The mean age of diabetes diagnosis within these families, was 43.4±16.8 years.

Ninety-seven (23.3%) diabetic individuals were considered to have advanced DN (proteinuria, *n* = 40, or ESRD, *n* = 57) while 312 (75.0%) were classified as non-DN controls. To improve power to detect significant associations between DN and SNPs identified in the GoKinD collections, we also expanded our nephropathy phenotype to include individuals with less severe nephropathy. For these comparisons, dichotomized cases included 28 additional individuals with high microalbuminuria. Seven diabetic individuals did not have ACR data available for classification of their nephropathy status or for the quantitative analysis of this trait; these individuals were excluded from all analyses. Proteinuria developed in 3 (0.8%) non-diabetic individuals. Clinical characteristics for examined members of the Joslin Study of Genetics of Nephropathy in Type 2 Diabetes Family Collection included in this study are provided in Table 1.

**Table 1:** Clinical characteristics of 798 examined members from 66 families from the Joslin Study of Genetics of Nephropathy in Type 2 Diabetes Family Collection.

Clinical characteristic	Non-diabetic individuals	Diabetic individuals
*n*	382	416
Men (%)	43.7	45.2
Age (years)	46.7±17.2	57.5±15.7
Age of diabetes diagnosis (years)	---	43.4±16.8
Duration of diabetes (years)	---	14.1±11.8
Diabetes treatment (%)		
Insulin only	---	40.6
Insulin and oral agents	---	6.0
Oral agents only	---	35.8
Diet	---	17.6
Treatment with ACE inhibitors (%)	3.7	18.8
BMI (kg/m^2^)	27.5±5.6	30.1±6.7
HbA_1c_ (%)	5.5±0.5	7.6±1.5
Systolic blood pressure (mmHg)	121.5±18.0	137.3±20.4
Diastolic blood pressure (mmHg)	77.2±10.1	78.2±10.3
Treatment with antihypertensive medication (%)	14.0	45.0
Treatment with ACE inhibitors (%)	3.7	18.8
ACR ( µg/mg), median (25^th^ and 75^th^ percentiles)	6.0 (4.0, 9.0)	17.0 (7.0, 237.2)*
Patients with microalbuminuria (%)	22 (5.8)	72 (17.3)
Patients with high microalbuminuria† (%)	7 (1.8)	28 (6.7)
Patients with proteinuria (%)	3 (0.8)	40 (9.6)
Patients with ESRD (%)	---	57‡ (13.7)

Baseline clinical characteristics are presented as mean values ± standard deviation.

HbA_1c_, glycosylated hemoglobin. ESRD, end-stage renal disease.

* ESRD patients were assigned ACR values of 3500 µg/mg.

† High microalbuminuria was defined as an ACR between 100 and 300 µg/mg.

‡ ESRD status was updated for members of this collection through the United States Renal Data System as of August 2008.

The distribution of relative pairs in the Joslin Study of Genetics of Nephropathy in Type 2 Diabetes Family Collection based on their relationship and DN status are provided in Table 2. These 66 extended families generated a total of 6,421 relative pairs; 1,026 of whom are concordant for diabetes. The diabetic relative pairs include 53 relative pairs that are concordant for advanced DN, 239 relative pairs that are disconcordant for advanced DN, 88 relative pairs concordant for DN, and 329 relative pairs disconcordant for DN. Additionally, there are 15 relative pairs concordant for ESRD and 134 relative pairs disconcordant for this phenotype.

**Table 2:** Summary of the relative pairs in the 66 families from the Joslin Study of Genetics of Nephropathy in Type 2 Diabetes Family Collection according to diabetes and nephropathy status.

			Advanced DN		DN		ESRD	
Relationship	All pairs	All diabetic pairs	Concordant pairs	Discordant pairs	Concordant pairs	Discordant pairs	Concordant pairs	Discordant pairs
Sib-pairs	1127	416	21	106	33	139	6	57
Half-sibs	51	14	1	1	1	1	0	1
Cousins	862	164	12	42	18	57	2	19
Parent-child	1674	135	8	29	11	44	4	19
Grandparent-Grandchild	1148	20	0	6	0	6	0	4
Avuncular	1559	277	11	55	25	82	3	34
All pairs	6421	1026	53	239	88	329	15	134

Advanced DN  =  diabetic individuals with proteinuria or ESRD; DN  =  diabetic individuals with high microalbuminuria, proteinuria, or ESRD.

No deviation from Hardy-Weinberg equilibrium was observed among the 13 SNPs included in this study in the entire collection or in analyses performed separately in affected and unaffected individuals (*P*>0.05).

Family-based association analyses of 6 SNPs across the four loci identified in the GoKinD GWA scan were performed in diabetic relatives as well as in all relatives combined. Among diabetic family members, rs1888747 on chromosome 9q21.32 showed evidence of association with advanced nephropathy among diabetic family members (affecteds only: *P* = 0.029 [adjusted *P* = 0.174], Z = 2.18; affecteds and unaffecteds: *P* = 0.037 [adjusted *P* = 0.222], Z = 2.08, Table 3). When we expanded our definition of DN to include individuals with high microalbuminuria, the strength of this association improved significantly (affecteds only: *P* = 1.74×10^−3^ [adjusted *P* = 0.010], Z = 3.13; affecteds and unaffecteds: *P* = 1.42×10^−3^ [adjusted *P* = 0.009], Z = 3.19, Table 4). In both comparisons, the associations on 9q21.32 were in the same direction as initially reported in the GoKinD collections. Although no other SNPs achieved statistical significance, variants on 7p14.3 and 13q33.3 shared directionality with associations identified in GoKinD (Table 3 and Table 4). Among all family members, only rs1888747 was nominally associated with nephropathy in analyses that included high microalbuminurics (affecteds only: *P* = 0.026 [adjusted *P* = 0.156], Z = 2.23; affecteds and unaffecteds: *P* = 0.017 [adjusted *P* = 0.102], Z = 2.38, Table S1 and Table S2). Lastly, dichotomous analyses restricted to diabetic family members with ESRD showed modest evidence of a statistical association with rs1888747 (affecteds only: *P* = 0.036, Z = 2.09; affecteds and unaffecteds: *P* = 0.046, Z = 2.00, Table 5); this SNP, however, did not achieve statistical significance when a conservative Bonferroni correction was applied (adjusted *P*>0.05).

**Table 3:** Family-based association results between DN-associated SNPs and advanced nephropathy (normoalbuminuria vs. proteinuria/ESRD) among diabetic family members.

				*Affecteds Only*					*Affecteds and Unaffecteds*				
SNP (risk allele)*	Chr.	Allele	Allele Frequency	# Families	S-E(S)	Var(S)	Z score	*P*-value (adjusted *P*-value)	# Families	S-E(S)	Var(S)	Z score	*P*-value (adjusted *P*-value)
rs39075 (G)	7p14.3	G	0.554	50	12.14	61.68	1.55	0.122	56	8.39	53.18	1.15	0.250
		A	0.446	50	−12.14	61.68	−1.55	(0.732)	56	−8.39	53.18	−1.15	(1.00)
rs1888747 (G)	9q21.32	G	0.690	49	17.84	67.03	2.18	0.029	54	14.99	51.80	2.08	0.037
		C	0.310	49	−17.84	67.03	−2.18	(0.174)	54	−14.99	51.80	−2.08	(0.222)
rs10868025 (A)	9q21.32	A	0.601	43	11.64	53.54	1.59	0.112	49	10.89	46.71	1.59	0.111
		G	0.399	43	−11.64	53.54	−1.59	(0.672)	49	−10.89	46.71	−1.59	(0.666)
rs451041 (A)	11p15.4	A	0.561	47	2.31	56.15	0.31	0.758	52	−0.24	53.20	−0.03	0.974
		G	0.439	47	−2.31	56.15	−0.31	(1.00)	52	0.24	53.20	0.03	(1.00)
rs1411766 (A)	13q33.3	G	0.598	52	−11.97	71.30	−1.42	0.156	55	−8.37	60.01	−1.08	0.280
		A	0.402	52	11.97	71.30	1.42	(0.936)	55	8.37	60.01	1.08	(1.00)
rs9521445 (A)	13q33.3	A	0.548	44	9.65	57.56	1.27	0.204	50	5.90	58.51	0.77	0.441
		C	0.452	44	−9.65	57.56	−1.27	(1.00)	50	−5.90	58.51	−0.77	(1.00)

# Families  =  number of nuclear families informative for the FBAT analysis.

S-E(S)  =  observed minus the expected transmission for each allele.

Var(S)  =  variance of the observed transmission for each allele.

Z score: positive values indicate risk alleles (i.e., increased transmission to affected individuals), negative values indicate protective alleles (i.e., reduced transmission to affected individuals).

* Risk allele reported in *Pezzolesi et al*. [18]

**Table 4:** Family-based association results between DN-associated SNPs and nephropathy (normoalbuminuria vs. high microalbuminuria/proteinuria/ESRD) among diabetic family members.

				*Affecteds Only*					*Affecteds and Unaffecteds*				
SNP (risk allele)*	Chr.	Allele	Allele Frequency	# Families	S-E(S)	Var(S)	Z score	*P*-value (adjusted *P*-value)	# Families	S-E(S)	Var(S)	Z score	*P*-value (adjusted *P*-value)
rs39075 (G)	7p14.3	G	0.554	51	9.44	75.06	1.09	0.276	57	5.67	72.49	0.67	0.504
		A	0.446	51	−9.44	75.06	−1.09	(1.00)	57	−5.67	72.49	−0.67	(1.00)
**rs1888747** (G)	**9q21.32**	**G**	**0.690**	**47**	**26.63**	**72.33**	**3.13**	**1.74×10^−3^**	**53**	**23.78**	**55.52**	**3.19**	**1.42×10^−3^**
		**C**	**0.310**	**47**	−**26.63**	**72.33**	−**3.13**	**(0.010)**	**53**	−**23.78**	**55.52**	−**3.19**	**(0.009)**
**rs10868025** (A)	**9q21.32**	**A**	**0.601**	**44**	**21.51**	**61.66**	**2.74**	**6.17×10^−3^**	**49**	**20.76**	**51.83**	**2.88**	**3.94×10^−3^**
		**G**	**0.399**	**44**	**−21.51**	**61.66**	**−2.74**	**(0.037)**	**49**	**−20.76**	**51.83**	**−2.88**	**(0.024)**
rs451041(A)	11p15.4	A	0.561	48	2.79	56.49	0.37	0.711	54	0.24	54.55	0.03	0.975
		G	0.439	48	**−**2.79	56.49	**−**0.37	(1.00)	54	**−**0.24	54.55	**−**0.03	(1.00)
rs1411766(A)	13q33.3	G	0.598	53	**−**4.06	64.70	**−**0.51	0.614	55	**−**0.46	54.62	**−**0.06	0.950
		A	0.402	53	4.06	64.70	0.51	(1.00)	55	0.46	54.62	0.06	(1.00)
rs9521445(A)	13q33.3	A	0.548	44	8.54	62.44	1.08	0.280	51	4.79	63.44	0.60	0.548
		C	0.452	44	**−**8.54	62.44	**−**1.08	(1.00)	51	**−**4.79	63.44	**−**0.60	(1.00)

# Families  =  number of nuclear families informative for the FBAT analysis.

S-E(S)  =  observed minus the expected transmission for each allele.

Var(S)  =  variance of the observed transmission for each allele.

Z score: positive values indicate risk alleles (i.e., increased transmission to affected individuals), negative values indicate protective alleles (i.e., reduced transmission to affected individuals).

Associations achieving nominal significance (*P*-value<0.05) are indicated in bold.

* Risk allele reported in *Pezzolesi et al*. [18]

**Table 5:** Family-based association results between DN-associated SNPs and ESRD among diabetic family members.

				*Affecteds Only*					*Affecteds and Unaffecteds*				
SNP (risk allele)*	Chr.	Allele	Allele Frequency	# Families	S-E(S)	Var(S)	Z score	*P*-value (adjusted *P*-value)	# Families	S-E(S)	Var(S)	Z score	*P*-value (adjusted *P*-value)
rs39075 (G)	7p14.3	G	0.554	52	5.01	45.24	0.75	0.456	58	1.26	49.16	0.18	0.857
		A	0.446	52	−5.01	45.24	−0.75	(1.00)	58	−1.26	49.16	−0.18	(1.00)
rs1888747 (G)	9q21.32	G	0.690	48	16.71	63.76	2.09	0.036	55	13.86	48.27	2.00	0.046
		C	0.310	48	−16.71	63.76	−2.09	(0.216)	55	−13.86	48.27	−2.00	(0.276)
rs10868025 (A)	9q21.32	A	0.601	44	6.44	50.27	0.91	0.364	50	5.69	42.88	0.87	0.385
		G	0.399	44	−6.44	50.27	−0.91	(1.00)	50	−5.69	42.88	−0.87	(1.00)
rs451041 (A)	11p15.4	A	0.561	45	6.85	42.11	1.06	0.291	50	4.30	35.52	0.72	0.471
		G	0.439	45	−6.85	42.11	−1.06	(1.00)	50	−4.30	35.52	−0.72	(1.00)
rs1411766 (A)	13q33.3	G	0.598	49	−8.38	50.10	−1.18	0.236	53	−4.78	44.69	−0.72	0.474
		A	0.402	49	8.38	50.10	1.18	(1.00)	53	4.78	44.69	0.72	(1.00)
rs9521445 (A)	13q33.3	A	0.548	44	11.22	47.27	1.63	0.103	49	7.47	47.83	1.08	0.280
		C	0.452	44	−11.22	47.27	−1.63	(0.618)	49	−7.47	47.83	−1.08	(1.00)

# Families  =  number of nuclear families informative for the FBAT analysis.

S-E(S)  =  observed minus the expected transmission for each allele.

Var(S)  =  variance of the observed transmission for each allele.

Z score: positive values indicate risk alleles (i.e., increased transmission to affected individuals), negative values indicate protective alleles (i.e., reduced transmission to affected individuals).

* Risk allele reported in *Pezzolesi et al*. [18]

In quantitative trait analyses, rs1888747 on chromosome 9q21.32 was similarly shown to be associated with logACR among diabetic family members (*P* = 0.030 [adjusted *P* = 0.180], Z = 2.17, Table 6) and in analyses extended to all family members in the Joslin Study of Genetics of Nephropathy in Type 2 Diabetes Family Collection (*P* = 0.017 [adjusted *P* = 0.102], Z = 2.39, Table S3). Controlling for reported ACE inhibitor treatment weakened the association between rs1888747 and albuminuria among diabetic family members and all family members (*P* = 0.217 [adjusted *P* = 1.00], Z = 1.24 and *P* = 0.121 [adjusted *P* = 0.726], Z = 1.55, respectively) while associations at rs10868025 (also located on chromosome 9q21.32) improved (diabetic family members: *P* = 0.012 [adjusted *P* = 0.072], Z = 2.50; all family members: *P* = 0.033 [adjusted *P* = 0.198], Z = 2.13).

**Table 6:** Family-based association analysis between DN-associated SNPs and logACR among diabetic family members.

SNP (risk allele)*	Chr.	Allele	Allele Frequency	# Families	S-E(S)	Var(S)	Z score	*P*-value (adjusted *P*-value)
rs39075 (G)	7p14.3	G	0.554	58	36.71	872.53	1.24	0.214
		A	0.446	58	−36.71	872.53	−1.24	(1.00)
rs1888747 (G)	9q21.32	G	0.690	54	54.68	636.86	2.17	0.030
		C	0.310	54	−54.68	636.86	−2.17	(0.180)
rs10868025 (A)	9q21.32	A	0.601	53	38.16	528.18	1.66	0.097
		G	0.399	53	−38.16	528.18	−1.66	(0.582)
rs451041 (A)	11p15.4	A	0.561	55	11.42	640.33	0.45	0.652
		G	0.439	55	−11.42	640.33	−0.45	(1.00)
rs1411766 (A)	13q33.3	G	0.598	55	−32.54	834.93	−1.13	0.260
		A	0.402	55	32.54	834.93	1.13	(1.00)
rs9521445 (A)	13q33.3	A	0.548	51	16.93	716.00	0.63	0.527
		C	0.452	51	−16.93	716.00	−0.63	(1.00)

# Families  =  number of nuclear families informative for the FBAT analysis.

S-E(S)  =  observed minus the expected transmission for each allele.

Var(S)  =  variance of the observed transmission for each allele.

Z score: positive values indicate risk alleles, negative values indicate protective alleles.

* Risk allele reported in *Pezzolesi et al*. [18]

No additional associations were observed between the remaining GoKinD SNPs and logACR in analyses either restricted to diabetic individuals or in those extended to include all available family members.

To further examine the susceptibility loci identified in GoKinD, we genotyped haplotype tagging SNPs across each locus and performed family-based multi-marker analyses for each nephropathy phenotype using the HBAT procedure in FBAT. Haplotypes formed by genotyped SNPs on chromosomes 7p14.3, 11p15.4, and 13q33.3 were not associated with any of the examined nephropathy phenotypes in analyses of diabetic individuals or in those performed in all family members (global *P*>0.05, data not shown). In contrast, haplotypes on chromosome 9q21.32 showed evidence of association with advanced nephropathy, nephropathy and logACR among diabetic family members (Table 7 and Table 8). While no haplotype on 9q21.32 was more strongly associated with nephropathy or logACR than the individual SNPs at this locus, the GTA haplotype, which is comprised of the risk alleles for both rs1888747 and rs10868025, is more strongly associated with an increased risk of advanced nephropathy than any individual SNP at this locus (affecteds only: *P* = 0.012, Z = 2.50; affecteds and unaffecteds: *P* = 0.018, Z = 2.37, Table 7). None of the genotyped haplotype tagging SNPs were associated with nephropathy in single marker analyses (Tables S4 through S9).

**Table 7:** Family-based haplotype analysis between chromosome 9q21.32* haplotypes and advanced nephropathy (normoalbuminuria vs. proteinuria/ESRD) and nephropathy (normoalbuminuria vs. high microalbuminuria/proteinuria/ESRD) among diabetic family members.

Advanced Nephropathy:		*Affecteds Only*					*Affecteds and Unaffecteds*				
Haplotype	Estimated Frequency	# Families	S-E(S)	Var(S)	Z score	*P*-value	# Families	S-E(S)	Var(S)	Z score	*P*-value
**GTA**	**0.437**	**54**	**20.18**	**65.00**	**2.50**	**0.012**	**59**	**16.56**	**48.98**	**2.37**	**0.018**
**CTG**	**0.310**	**50**	**−17.31**	**55.12**	**−2.33**	**0.020**	**55**	**−16.23**	**41.24**	**−2.53**	**0.011**
GGA	0.163	39	−8.94	41.87	−1.38	0.167	43	−4.71	26.45	−0.92	0.359
GTG	0.088	29	6.09	18.77	1.41	0.160	33	4.40	14.92	1.14	0.254
				Global *P*-value		**0.049**			Global *P*-value		**0.034**

Nephropathy:		*Affecteds Only*					*Affecteds and Unaffecteds*				
Haplotype	Estimated Frequency	# Families	S-E(S)	Var(S)	Z score	*P*-value	# Families	S-E(S)	Var(S)	Z score	*P*-value
**GTA**	**0.437**	**55**	**27.16**	**82.39**	**2.99**	**2.77×10^−3^**	**59**	**23.54**	**67.72**	**2.86**	**4.24×10^−3^**
**CTG**	**0.310**	**48**	−**23.97**	**72.37**	−**2.82**	**4.84×10^−3^**	**55**	−**22.89**	**57.36**	−**3.02**	**2.51×10^−3^**
GGA	0.163	39	−7.10	47.35	−1.03	0.303	43	−2.87	33.64	−0.49	0.621
GTG	0.088	32	3.92	19.03	0.90	0.369	34	2.24	16.03	0.56	0.577
				Global *P*-value		**0.012**			Global *P*-value		**0.013**

* 9q21.32 haplotypes: rs1888747, rs1929547, and rs10868025.

Haplotypes with estimated frequencies ≥0.01 are provided and were used to calculate global *P*-values.

# Families  =  number of nuclear families informative for the HBAT analysis; a minimum of 5 informative families for each haplotype was required to compute global tests.

S-E(S)  =  observed minus the expected transmission for each haplotype.

Var(S)  =  variance of the observed transmission for each haplotype.

Z score: positive values indicate risk haplotypes, negative values indicate protective haplotypes.

Associations achieving nominal significance (*P*-value<0.05) are indicated in bold.

**Table 8:** Family-based haplotype analysis between chromosome 9q21.32* haplotypes and logACR among diabetic family members.

Haplotype	Estimated Frequency	# Families	S-E(S)	Var(S)	Z score	*P*-value
**GTA**	**0.437**	**62**	**63.05**	**904.53**	**2.10**	**0.036**
CTG	0.310	54	−46.27	743.97	−1.70	0.090
GGA	0.163	42	−28.61	825.82	−1.00	0.320
GTG	0.088	34	11.89	276.38	0.72	0.474
				Global *P*-value		0.192

* 9q21.32 haplotypes: rs1888747, rs1929547, and rs10868025.

Haplotypes with estimated frequencies ≥0.01 are provided and were used to calculate global *P*-values.

# Families  =  number of nuclear families informative for the HBAT analysis; a minimum of 5 informative families for each haplotype was required to compute global tests.

S-E(S)  =  observed minus the expected transmission for each haplotype.

Var(S)  =  variance of the observed transmission for each haplotype.

Z score: positive values indicate risk haplotypes, negative values indicate protective haplotypes.

Associations achieving nominal significance (*P*-value<0.05) are indicated in bold.

## Discussion

Our GWA scan of the GoKinD collections identified strong association at four distinct chromosomal regions, including loci on chromosomes 9q21.32, 11p15.4, and 13q33.3 that have since been confirmed in multiple collections comprised of unrelated T1D or T2D subjects [18], [20]–[22]. In the present report, we extend these findings further by providing additional support for associations at chromosome 9q21.32 in a large collection of related T2D patients from the Joslin Study of Genetics of Nephropathy in Type 2 Diabetes Family Collection. In this study, a statistically significant association was observed between this locus and the risk of high microalbuminuria, proteinuria, and ESRD among diabetic individuals in these families.

DN is well-recognized to be a complex disease, characterized by both abnormalities in urinary albumin excretion and declining renal function. While most patients with DN exhibit some degree of elevated urinary albumin excretion, for some, this abnormality is concurrent with impaired renal function; a subset of these individuals eventually require renal replacement therapy. This phenotypic heterogeneity suggests that multiple factors, some genetic and some non-genetic, contribute to the distinct stages of DN. In the present study, we report evidence of association at the 9q21.32 locus with advanced DN (i.e., proteinuria and ESRD). The strength of these associations improved significantly when we expanded our nephropathy phenotype to include individuals with less severe DN (i.e., patients with high microalbuminuria, ACR values >100 µg/mg). Based on these findings, we hypothesize that variation at this locus contributes to the early stages of nephropathy in diabetes. Furthermore, we hypothesize that other genetic factors are likely involved in the progression of DN and the decline in renal function that accompanies the latter stages of this disease process.

Associations at common variants on the 9q21.32 locus have now been confirmed in four distinct collections; T1D patients from the GoKinD collections, the Joslin Study of Genetics of Nephropathy in Type 2 Diabetes Family Collection, Caucasian participants from the Diabetes Control and Complications Trial/Epidemiology of Diabetes Interventions and Complications (DCCT/EDIC) study [18], and African Americans with ESRD due to T2D [21]. In addition to these studies, a strong protective affect of the rs1888747 DN-risk allele observed in populations of European ancestry has recently been reported in unrelated Japanese T2D patients with microalbuminuria [20]; a notable finding that may be attributed to allelic heterogeneity resulting from the ancestral backgrounds of these two ethnic groups. Despite being quite underpowered, in this study Maeda et al. identified a strong association at rs1888747 in a comparison of only 32 microalbuminuric patients who progressed to overt proteinuria and 168 individuals who remained microalbuminuric (i.e., non-progressors).

Additional support for a role of this locus in nephropathy comes from a genetic study of albuminuria quantitative trait loci (QTL) performed in an intercross of albuminuria resistant and susceptible mouse strains [28]. Using this approach, Sheehan et al. were able to localize a QTL associated with increased urinary albumin on mouse chromosome 4, a region homologous to the DN-associated locus on chromosome 9q21.32 seen in human populations. This striking concordance between human and mouse suggests that a common disease mechanism may link these renal damage phenotypes.

A major challenge in dissecting the genetic basis of complex traits, including DN, is that many of the common variants that have been reproducibly shown to be associated with disease explain only a modest proportion of the overall risk of disease. Although less powerful than population-based designs, family-based approaches such as the one employed in our study will prove crucial in uncovering variants that have much larger contributions to the genetic basis of disease as rare variants that are expected to explain a larger proportion of the heritability of a given phenotype are enrich in related individuals.

The present study has modest power (<80%) to detect similar effects as those observed by Pezzolesi et al. [18]. Despite this limitation, our analysis increases support that previously reported associations at the 9q21.32 locus are genuine diabetic nephropathy susceptibility loci and, given the limited power of our study, suggest that the true effect sizes attributed to variants at this locus may in fact be larger than previously estimated. More specifically, the associations identified in the GoKinD collections at the 9q21.32 locus and confirmed in the Joslin Study of Genetics of Nephropathy in Type 2 Diabetes Family Collection occur at common SNPs (risk allele frequency ≈ 60–70%) of modest effect (odds ratio = 1.40–1.50). It remains possible that this association is due to the presence of a ‘synthetic association’ caused by one or more rare variants located some distance from the observed associations [29]. Carriers of the rs1888744 risk allele in our families are potential future candidates for targeted next-generation sequencing of this locus to identify rare variants that may explain a substantial proportion of the heritability of DN risk observed in these families.

In contrast to studies of unrelated cases and controls, family-based designs, such as the one used in the present study, are robust to population admixture and stratification. Additionally, family members also tend to have more homogeneity of environmental factors that could confound genetic associations with the phenotype of interest. Despite these advantages, we acknowledge that the present study is not without its limitations. Patients in Joslin Study of Genetics of Nephropathy in Type 2 Diabetes Family Collection were recruited irrespective of their nephropathy status and their phenotypic characteristics were primarily derived from baseline data taken at the time of enrollment. Understanding the natural history of diabetic nephropathy and recognizing the limitations of this cross-sectional assessment of kidney phenotypes, we chose to use the USRDS database to track individuals that progressed to ESRD. Individuals who might have progressed to ESRD but refused renal replacement therapy are not represented in USRDS and, depending on their renal status at baseline, may have misclassification of their renal phenotype. In lieu of longitudinal follow-up of all members of the Joslin Study of Genetics of Nephropathy in Type 2 Diabetes Family Collection, we recognize that the potential misclassification of these individuals is a limitation of our study. Importantly, however, we note that any phenotypic misclassification due to our inability to track participants who refused renal replacement therapy is independent of their carrier status of DN risk and/or non-risk alleles; the resulting non-differential misclassification could only have biased our findings toward the null. A second limitation worthy of discussion is the potential competing risk of death prior to the development of ESRD. Because of the high rate of mortality experienced by diabetic patients with ESRD, variants associated with this phenotype may alternatively be associated with survival in the presence of severe kidney disease. Although the present study lacks the power to formally assess whether the effects of variants at the 9q21.32 locus differ according to duration of ESRD, we have previously shown that the odds ratios for these variants are consistent across tertiles of ESRD duration in patients with T1D and ESRD, suggesting that these associations are not due to survival bias [18].

rs1888747 lies approximately 2 kilo-basepair upstream of *FRMD3*, a gene that we have previously shown to be expressed in both human kidney mesangial cells and proximal tubular cells [18]. Additional work has further demonstrated that *FRMD3* is also expressed in human podocytes and that its protein product, the 4.1O protein, interacts with nephrin, podocin, and actin, suggesting this protein is involved in maintaining the function and integrity of the slit diaphragm (unpublished data). Most recently, we have shown that rs1888747's risk allele generates a transcription factor binding site in a repressive promote module that is shared by multiple members of the bone morphogenetic protein (*BMP*) signaling pathway; a pathway that has previously been implicated in the development of DN [30], [31]. Hierarchical clustering of expression data for *FRMD3* and its co-expressed transcripts suggests that these genes are linked to early progression in DN [30]. Coupled with the strong association we observed at this locus in diabetic family members with less severe DN in the present study, we hypothesize that the 9q21.32 locus contributes to glomerular injury early in DN's pathogenesis.

In summary, our study provides further evidence that the 9q21.32 region is a susceptibility locus for DN. Coupled with its proximity to the association at this locus, *FRMD3* appears to be both a strong positional and biological candidate gene for DN.

## Supporting Information

Table S1 Family-based association results between DN-associated SNPs and advanced nephropathy (normoalbuminuria vs. proteinuria/ESRD) among all family members.
(DOC)

Table S2 Family-based association results between DN-associated SNPs and nephropathy (normoalbuminuria vs. high microalbuminuria/proteinuria/ESRD) among all family members.
(DOC)

Table S3 Family-based association analysis between DN-associated SNPs and logACR among all family members.
(DOC)

Table S4 Single marker family-based association analyses between haplotype tagging SNPs across the four GoKinD loci and advanced nephropathy among diabetic family members. Affecteds and unaffecteds analyses are presented.
(DOC)

Table S5 Single marker family-based association analyses between haplotype tagging SNPs across the four GoKinD loci and nephropathy among diabetic family members. Affecteds and unaffecteds analyses are presented.
(DOC)

Table S6 Single marker family-based association analyses between haplotype tagging SNPs across the four GoKinD loci and logACR among diabetic family members. Affecteds and unaffecteds analyses are presented.
(DOC)

Table S7 Single marker family-based association analyses between haplotype tagging SNPs across the four GoKinD loci and advanced nephropathy among all family members. Affecteds and unaffecteds analyses are presented.
(DOC)

Table S8 Single marker family-based association analyses between haplotype tagging SNPs across the four GoKinD loci and nephropathy among all family members. Affecteds and unaffecteds analyses are presented.
(DOC)

Table S9 Single marker family-based association analyses between haplotype tagging SNPs across the four GoKinD loci and logACR among all family members. Affecteds and unaffecteds analyses are presented.
(DOC)
