# Supplementary material for: Family-Based Association Analysis Confirms the Role of the Chromosome 9q21.32 Locus in the Susceptibility of Diabetic Nephropathy
Source: PLoS One. 2013 Mar 29;8(3):e60301. doi: 10.1371/journal.pone.0060301 (PMC3612041; doi:10.1371/journal.pone.0060301)
Supplement: Table S1 — Family-based association results between DN-associated SNPs and advanced nephropathy (normoalbuminuria vs. proteinuria/ESRD) among all family members. (DOC) [file pone.0060301.s001.doc]

| **Table S1.** Family-based association results between DN-associated SNPs and advanced nephropathy (normoalbuminuria vs. proteinuria/ESRD) among all family members. | | | | | | | | | | | | | |
| --- | --- | --- | --- | --- | --- | --- | --- | --- | --- | --- | --- | --- | --- |
|  |  |  |  | *Affecteds Only* | | | | | *Affecteds and Unaffecteds* | | | | |
| SNP  (risk allele)* | Chr. | Allele | Allele Frequency | # Families | S-E(S) | Var(S) | Z score | *P*-value  (adjusted *P*-value) | # Families | S-E(S) | Var(S) | Z score | *P*-value  (adjusted *P*-value) |
| rs39075 (G) | 7p14.3 | G | 0.554 | 53 | 11.78 | 101.53 | 1.17 | 0.242 | 53 | 8.03 | 66.55 | 0.98 | 0.325 |
|  |  | A | 0.446 | 53 | -11.78 | 101.53 | -1.17 | (1.00) | 53 | -8.03 | 66.55 | -0.98 | (1.00) |
| rs1888747 (G) | 9q21.32 | G | 0.690 | 49 | 15.68 | 110.91 | 1.49 | 0.136 | 50 | 12.83 | 74.78 | 1.48 | 0.138 |
|  |  | C | 0.310 | 49 | -15.68 | 110.91 | -1.49 | (0.816) | 50 | -12.83 | 74.78 | -1.48 | (0.828) |
| rs10868025 (A) | 9q21.32 | A | 0.601 | 46 | 8.86 | 72.59 | 1.04 | 0.299 | 48 | 8.11 | 50.97 | 1.14 | 0.256 |
|  |  | G | 0.399 | 46 | -8.86 | 72.59 | -1.04 | (1.00) | 48 | -8.11 | 50.97 | -1.14 | (1.00) |
| rs451041 (A) | 11p15.4 | A | 0.561 | 48 | 6.08 | 79.61 | 0.68 | 0.496 | 51 | 3.53 | 50.47 | 0.50 | 0.619 |
|  |  | G | 0.439 | 48 | -6.08 | 79.61 | -0.68 | (1.00) | 51 | -3.53 | 50.47 | -0.50 | (1.00) |
| rs1411766 (A) | 13q33.3 | G | 0.598 | 52 | -14.25 | 94.55 | -1.47 | 0.143 | 54 | -10.65 | 65.61 | -1.32 | 0.188 |
|  |  | A | 0.402 | 52 | 14.25 | 94.55 | 1.47 | (0.858) | 54 | 10.65 | 65.61 | 1.32 | (1.00) |
| rs9521445 (A) | 13q33.3 | A | 0.548 | 44 | 10.33 | 57.08 | 1.37 | 0.172 | 48 | 6.58 | 42.96 | 1.00 | 0.316 |
|  |  | C | 0.452 | 44 | -10.33 | 57.08 | -1.37 | (1.00) | 48 | -6.58 | 42.96 | -1.00 | (1.00) |

# Families = number of nuclear families informative for the FBAT analysis

S-E(S) = observed minus the expected transmission for each allele

Var(S) = variance of the observed transmission for each allele

Z score: positive values indicate risk alleles (i.e., increased transmission to affected individuals), negative values indicate protective alleles (i.e., reduced transmission to affected individuals)

*Risk allele reported in *Pezzolesi et al.* [18]
