# Supplementary material for: Family-Based Association Analysis Confirms the Role of the Chromosome 9q21.32 Locus in the Susceptibility of Diabetic Nephropathy
Source: PLoS One. 2013 Mar 29;8(3):e60301. doi: 10.1371/journal.pone.0060301 (PMC3612041; doi:10.1371/journal.pone.0060301)
Supplement: Table S2 — Family-based association results between DN-associated SNPs and nephropathy (normoalbuminuria vs. high microalbuminuria/proteinuria/ESRD) among all family members. (DOC) [file pone.0060301.s002.doc]

| **Table S2.** Family-based association results between DN-associated SNPs and nephropathy (normoalbuminuria vs. high microalbuminuria/proteinuria/ ESRD) among all family members. | | | | | | | | | | | | | |
| --- | --- | --- | --- | --- | --- | --- | --- | --- | --- | --- | --- | --- | --- |
|  |  |  |  | *Affecteds Only* | | | | | *Affecteds and Unaffecteds* | | | | |
| SNP  (risk allele)* | Chr. | Allele | Allele Frequency | # Families | S-E(S) | Var(S) | Z score | *P*-value  (adjusted *P*-value) | # Families | S-E(S) | Var(S) | Z score | *P*-value  (adjusted *P*-value) |
| rs39075 (G) | 7p14.3 | G | 0.554 | 56 | 10.41 | 104.19 | 1.02 | 0.308 | 56 | 6.66 | 70.34 | 0.79 | 0.427 |
|  |  | A | 0.446 | 56 | -10.41 | 104.19 | -1.02 | (1.00) | 56 | -6.66 | 70.34 | -0.79 | (1.00) |
| rs1888747 (G) | 9q21.32 | G | 0.690 | 48 | 24.48 | 120.22 | 2.23 | 0.026 | 50 | 21.63 | 82.51 | 2.38 | 0.017 |
|  |  | C | 0.310 | 48 | -24.48 | 120.22 | -2.23 | (0.156) | 50 | -21.63 | 82.51 | -2.38 | (0.102) |
| rs10868025 (A) | 9q21.32 | A | 0.601 | 43 | 19.72 | 87.79 | 2.10 | 0.035 | 48 | 18.97 | 62.56 | 2.40 | 0.016 |
|  |  | G | 0.399 | 43 | -19.72 | 87.79 | -2.10 | (0.210) | 48 | -18.97 | 62.56 | -2.40 | (0.096) |
| rs451041 (A) | 11p15.4 | A | 0.561 | 48 | 4.56 | 72.05 | 0.54 | 0.591 | 51 | 2.01 | 43.91 | 0.30 | 0.76 |
|  |  | G | 0.439 | 48 | -4.56 | 72.05 | -0.54 | (1.00) | 51 | -2.01 | 43.91 | -0.30 | (1.00) |
| rs1411766 (A) | 13q33.3 | G | 0.598 | 52 | -6.34 | 89.49 | -0.67 | 0.502 | 54 | -2.74 | 61.75 | -0.35 | 0.727 |
|  |  | A | 0.402 | 52 | 6.34 | 89.49 | 0.67 | (1.00) | 54 | 2.74 | 61.75 | 0.35 | (1.00) |
| rs9521445 (A) | 13q33.3 | A | 0.548 | 45 | 10.22 | 60.39 | 1.32 | 0.189 | 49 | 6.47 | 46.03 | 0.95 | 0.340 |
|  |  | C | 0.452 | 45 | -10.22 | 60.39 | -1.32 | (1.00) | 49 | -6.47 | 46.03 | -0.95 | (1.00) |

# Families = number of nuclear families informative for the FBAT analysis

S-E(S) = observed minus the expected transmission for each allele

Var(S) = variance of the observed transmission for each allele

Z score: positive values indicate risk alleles (i.e., increased transmission to affected individuals), negative values indicate protective alleles (i.e., reduced transmission to affected individuals)

*Risk allele reported in *Pezzolesi et al.* [18]
