# Supplementary material for: Family-Based Association Analysis Confirms the Role of the Chromosome 9q21.32 Locus in the Susceptibility of Diabetic Nephropathy
Source: PLoS One. 2013 Mar 29;8(3):e60301. doi: 10.1371/journal.pone.0060301 (PMC3612041; doi:10.1371/journal.pone.0060301)
Supplement: Table S3 — Family-based association analysis between DN-associated SNPs and logACR among all family members. (DOC) [file pone.0060301.s003.doc]

**Table S3.** Family-based association analysis between DN-associated SNPs and logACR among all family members.

| SNP  (risk allele)* | Chr. | Allele | Allele Frequency | # Families | S-E(S) | Var(S) | Z score | *P*-value  (adjusted *P*-value) |
| --- | --- | --- | --- | --- | --- | --- | --- | --- |
| rs39075 (G) | 7p14.3 | G | 0.554 | 60 | 43.50 | 985.93 | 1.39 | 0.166 |
|  |  | A | 0.446 | 60 | -43.50 | 985.93 | -1.39 | (1.00) |
| rs1888747 (G) | 9q21.32 | G | 0.69 | 56 | 67.87 | 806.50 | 2.39 | 0.017 |
|  |  | C | 0.31 | 56 | -67.87 | 806.50 | -2.39 | (0.102) |
| rs10868025 (A) | 9q21.32 | A | 0.601 | 55 | 51.86 | 571.33 | 2.17 | 0.030 |
|  |  | G | 0.399 | 55 | -51.86 | 571.33 | -2.17 | (0.180) |
| rs451041 (A) | 11p15.4 | A | 0.561 | 57 | 8.30 | 531.89 | 0.36 | 0.719 |
|  |  | G | 0.439 | 57 | -8.30 | 531.89 | -0.36 | (1.00) |
| rs1411766 (A) | 13q33.3 | G | 0.598 | 57 | -18.60 | 800.09 | -0.66 | 0.511 |
|  |  | A | 0.402 | 57 | 18.60 | 800.09 | 0.66 | (1.00) |
| rs9521445 (A) | 13q33.3 | A | 0.548 | 55 | 20.90 | 607.43 | 0.85 | 0.396 |
|  |  | C | 0.452 | 55 | -20.90 | 607.43 | -0.85 | (1.00) |

# Families = number of nuclear families informative for the FBAT analysis

S-E(S) = observed minus the expected transmission for each allele

Var(S) = variance of the observed transmission for each allele

Z score: positive values indicate risk alleles, negative values indicate protective alleles

*Risk allele reported in *Pezzolesi et al.* [18]
