# Supplementary material for: Family-Based Association Analysis Confirms the Role of the Chromosome 9q21.32 Locus in the Susceptibility of Diabetic Nephropathy
Source: PLoS One. 2013 Mar 29;8(3):e60301. doi: 10.1371/journal.pone.0060301 (PMC3612041; doi:10.1371/journal.pone.0060301)
Supplement: Table S4 — Single marker family-based association analyses between haplotype tagging SNPs across the four GoKinD loci and advanced nephropathy among diabetic family members. Affecteds and unaffecteds analyses are presented. (DOC) [file pone.0060301.s004.doc]

**Table S4.** Single marker family-based association analyses between haplotype tagging SNPs across the four GoKinD loci and advanced nephropathy among diabetic family members. Affecteds and unaffecteds analyses are presented.

| SNP | Chr. | Allele | Allele Frequency | # Families | S-E(S) | Var(S) | Z score | *P*-value  (adjusted *P*-value) |
| --- | --- | --- | --- | --- | --- | --- | --- | --- |
| rs39077 | 7p14.3 | A | 0.616 | 51 | -7.64 | 43.74 | -1.16 | 0.248 |
|  |  | C | 0.384 | 51 | 7.64 | 43.74 | 1.16 | (1.00) |
| rs17679605 | 7p14.3 | T | 0.835 | 40 | -2.22 | 31.30 | -0.40 | 0.691 |
|  |  | C | 0.165 | 40 | 2.22 | 31.30 | 0.40 | (1.00) |
| rs1929547 | 9q21.32 | T | 0.825 | 42 | -0.90 | 25.98 | -0.18 | 0.860 |
|  |  | G | 0.175 | 42 | 0.90 | 25.98 | 0.18 | (1.00) |
| rs12793371 | 11p15.4 | A | 0.676 | 51 | -1.35 | 57.55 | -0.18 | 0.859 |
|  |  | G | 0.324 | 51 | 1.35 | 57.55 | 0.18 | (1.00) |
| rs417957 | 11p15.4 | A | 0.553 | 53 | 4.60 | 39.51 | 0.73 | 0.465 |
|  |  | G | 0.447 | 53 | -4.60 | 39.51 | -0.73 | (1.00) |
| rs9555618 | 13q33.3 | G | 0.565 | 55 | -10.13 | 74.74 | -1.17 | 0.241 |
|  |  | A | 0.435 | 55 | 10.13 | 74.74 | 1.17 | (1.00) |
| rs7989975 | 13q33.3 | A | 0.837 | 33 | 5.50 | 17.93 | 1.30 | 0.194 |
|  |  | C | 0.163 | 33 | -5.50 | 17.93 | -1.30 | (1.00) |
