# Supplementary material for: Family-Based Association Analysis Confirms the Role of the Chromosome 9q21.32 Locus in the Susceptibility of Diabetic Nephropathy
Source: PLoS One. 2013 Mar 29;8(3):e60301. doi: 10.1371/journal.pone.0060301 (PMC3612041; doi:10.1371/journal.pone.0060301)
Supplement: Table S5 — Single marker family-based association analyses between haplotype tagging SNPs across the four GoKinD loci and nephropathy among diabetic family members. Affecteds and unaffecteds analyses are presented. (DOC) [file pone.0060301.s005.doc]

**Table S5.** Single marker family-based association analyses between haplotype tagging SNPs across the four GoKinD loci and nephropathy among diabetic family members. Affecteds and unaffecteds analyses are presented.

| SNP | Chr. | Allele | Allele Frequency | # Families | S-E(S) | Var(S) | Z score | *P*-value  (adjusted *P*-value) |
| --- | --- | --- | --- | --- | --- | --- | --- | --- |
| rs39077 | 7p14.3 | A | 0.616 | 52 | -6.08 | 46.09 | -1.00 | 0.318 |
|  |  | C | 0.384 | 52 | 6.08 | 46.09 | 1.00 | (1.00) |
| rs17679605 | 7p14.3 | T | 0.835 | 40 | 1.44 | 34.48 | 0.25 | 0.806 |
|  |  | C | 0.165 | 40 | -1.44 | 34.48 | -0.25 | (1.00) |
| rs1929547 | 9q21.32 | T | 0.825 | 42 | -4.20 | 35.25 | -0.71 | 0.479 |
|  |  | G | 0.175 | 42 | 4.20 | 35.25 | 0.71 | (1.00) |
| rs12793371 | 11p15.4 | A | 0.676 | 52 | -2.81 | 57.98 | -0.37 | 0.712 |
|  |  | G | 0.324 | 52 | 2.81 | 57.98 | 0.37 | (1.00) |
| rs417957 | 11p15.4 | A | 0.553 | 53 | 6.12 | 40.04 | 0.97 | 0.334 |
|  |  | G | 0.447 | 53 | -6.12 | 40.04 | -0.97 | (1.00) |
| rs9555618 | 13q33.3 | G | 0.565 | 55 | -3.22 | 70.95 | -0.38 | 0.703 |
|  |  | A | 0.435 | 55 | 3.22 | 70.95 | 0.38 | (1.00) |
| rs7989975 | 13q33.3 | A | 0.837 | 32 | 1.84 | 18.28 | 0.43 | 0.667 |
|  |  | C | 0.163 | 32 | -1.84 | 18.28 | -0.43 | (1.00) |
