# Supplementary material for: Family-Based Association Analysis Confirms the Role of the Chromosome 9q21.32 Locus in the Susceptibility of Diabetic Nephropathy
Source: PLoS One. 2013 Mar 29;8(3):e60301. doi: 10.1371/journal.pone.0060301 (PMC3612041; doi:10.1371/journal.pone.0060301)
Supplement: Table S6 — Single marker family-based association analyses between haplotype tagging SNPs across the four GoKinD loci and logACR among diabetic family members. Affecteds and unaffecteds analyses are presented. (DOC) [file pone.0060301.s006.doc]

**Table S6.** Single marker family-based association analyses between haplotype tagging SNPs across the four GoKinD loci and logACR among diabetic family members. Affecteds and unaffecteds analyses are presented.

| SNP | Chr. | Allele | Allele Frequency | # Families | S-E(S) | Var(S) | Z score | *P*-value  (adjusted *P*-value) |
| --- | --- | --- | --- | --- | --- | --- | --- | --- |
| rs39077 | 7p14.3 | A | 0.616 | 53 | -40.71 | 621.38 | -1.63 | 0.102 |
|  |  | C | 0.384 | 53 | 40.71 | 621.38 | 1.63 | (0.612) |
| rs17679605 | 7p14.3 | T | 0.835 | 40 | -2.96 | 388.81 | -0.15 | 0.881 |
|  |  | C | 0.165 | 40 | 2.96 | 388.81 | 0.15 | (1.00) |
| rs1929547 | 9q21.32 | T | 0.825 | 42 | -5.35 | 454.23 | -0.25 | 0.802 |
|  |  | G | 0.175 | 42 | 5.35 | 454.23 | 0.25 | (1.00) |
| rs12793371 | 11p15.4 | A | 0.676 | 54 | 18.02 | 613.49 | 0.73 | 0.467 |
|  |  | G | 0.324 | 54 | -18.02 | 613.49 | -0.73 | (1.00) |
| rs417957 | 11p15.4 | A | 0.553 | 53 | 3.68 | 615.01 | 0.15 | 0.882 |
|  |  | G | 0.447 | 53 | -3.68 | 615.01 | -0.15 | (1.00) |
| rs9555618 | 13q33.3 | G | 0.565 | 56 | -29.76 | 1006.63 | -0.94 | 0.348 |
|  |  | A | 0.435 | 56 | 29.76 | 1006.63 | 0.94 | (1.00) |
| rs7989975 | 13q33.3 | A | 0.837 | 34 | 10.93 | 386.03 | 0.56 | 0.578 |
|  |  | C | 0.163 | 34 | -10.93 | 386.03 | -0.56 | (1.00) |

# Families = number of nuclear families informative for the FBAT analysis

S-E(S) = observed minus the expected transmission for each allele

Var(S) = variance of the observed transmission for each allele

Z score: positive values indicate risk alleles, negative values indicate protective alleles
