# Supplementary material for: Family-Based Association Analysis Confirms the Role of the Chromosome 9q21.32 Locus in the Susceptibility of Diabetic Nephropathy
Source: PLoS One. 2013 Mar 29;8(3):e60301. doi: 10.1371/journal.pone.0060301 (PMC3612041; doi:10.1371/journal.pone.0060301)
Supplement: Table S7 — Single marker family-based association analyses between haplotype tagging SNPs across the four GoKinD loci and advanced nephropathy among all family members. Affecteds and unaffecteds analyses are presented. (DOC) [file pone.0060301.s007.doc]

**Table S7.** Single marker family-based association analyses between haplotype tagging SNPs across the four GoKinD loci and advanced nephropathy among all family members. Affecteds and unaffecteds analyses are presented.

| SNP | Chr. | Allele | Allele Frequency | # Families | S-E(S) | Var(S) | Z score | *P*-value  (adjusted *P*-value) |
| --- | --- | --- | --- | --- | --- | --- | --- | --- |
| rs39077 | 7p14.3 | A | 0.616 | 47 | -5.85 | 56.86 | -0.78 | 0.438 |
|  |  | C | 0.384 | 47 | 5.85 | 56.86 | 0.78 | (1.00) |
| rs17679605 | 7p14.3 | T | 0.835 | 38 | -7.67 | 34.85 | -1.30 | 0.194 |
|  |  | C | 0.165 | 38 | 7.67 | 34.85 | 1.30 | (1.00) |
| rs1929547 | 9q21.32 | T | 0.825 | 41 | 5.30 | 33.32 | 0.92 | 0.359 |
|  |  | G | 0.175 | 41 | -5.30 | 33.32 | -0.92 | (1.00) |
| rs12793371 | 11p15.4 | A | 0.676 | 47 | 6.56 | 50.78 | 0.92 | 0.357 |
|  |  | G | 0.324 | 47 | -6.56 | 50.78 | -0.92 | (1.00) |
| rs417957 | 11p15.4 | A | 0.553 | 52 | -3.70 | 51.63 | -0.51 | 0.607 |
|  |  | G | 0.447 | 52 | 3.70 | 51.63 | 0.51 | (1.00) |
| rs9555618 | 13q33.3 | G | 0.565 | 53 | -10.96 | 77.09 | -1.25 | 0.212 |
|  |  | A | 0.435 | 53 | 10.96 | 77/09 | 1.25 | (1.00) |
| rs7989975 | 13q33.3 | A | 0.837 | 33 | 3.40 | 16.45 | 0.84 | 0.401 |
|  |  | C | 0.163 | 33 | -3.40 | 16.45 | -0.84 | (1.00) |
