# Supplementary material for: Family-Based Association Analysis Confirms the Role of the Chromosome 9q21.32 Locus in the Susceptibility of Diabetic Nephropathy
Source: PLoS One. 2013 Mar 29;8(3):e60301. doi: 10.1371/journal.pone.0060301 (PMC3612041; doi:10.1371/journal.pone.0060301)
Supplement: Table S8 — Single marker family-based association analyses between haplotype tagging SNPs across the four GoKinD loci and nephropathy among all family members. Affecteds and unaffecteds analyses are presented. (DOC) [file pone.0060301.s008.doc]

**Table S8.** Single marker family-based association analyses between haplotype tagging SNPs across the four GoKinD loci and nephropathy among all family members. Affecteds and unaffecteds analyses are presented.

| SNP | Chr. | Allele | Allele Frequency | # Families | S-E(S) | Var(S) | Z score | *P*-value  (adjusted *P*-value) |
| --- | --- | --- | --- | --- | --- | --- | --- | --- |
| rs39077 | 7p14.3 | A | 0.616 | 48 | -4.31 | 59.30 | -0.56 | 0.575 |
|  |  | C | 0.384 | 48 | 4.31 | 59.30 | 0.56 | (1.00) |
| rs17679605 | 7p14.3 | T | 0.835 | 38 | -4.01 | 31.71 | -0.71 | 0.477 |
|  |  | C | 0.165 | 38 | 4.01 | 31.71 | 0.71 | (1.00) |
| rs1929547 | 9q21.32 | T | 0.825 | 41 | 3.00 | 37.98 | 0.49 | 0.626 |
|  |  | G | 0.175 | 41 | -3.00 | 37.98 | -0.49 | (1.00) |
| rs12793371 | 11p15.4 | A | 0.676 | 48 | 4.09 | 51.11 | 0.57 | 0.567 |
|  |  | G | 0.324 | 48 | -4.09 | 51.11 | -0.57 | (1.00) |
| rs417957 | 11p15.4 | A | 0.553 | 52 | -1.17 | 44.73 | -0.18 | 0.861 |
|  |  | G | 0.447 | 52 | 1.17 | 44.73 | 0.18 | (1.00) |
| rs9555618 | 13q33.3 | G | 0.565 | 53 | -3.05 | 69.16 | -0.37 | 0.714 |
|  |  | A | 0.435 | 53 | 3.05 | 69.16 | 0.37 | (1.00) |
| rs7989975 | 13q33.3 | A | 0.837 | 32 | -1.26 | 15.55 | -0.32 | 0.749 |
|  |  | C | 0.163 | 32 | 1.26 | 15.55 | 0.32 | (1.00) |
