# Supplementary material for: Family-Based Association Analysis Confirms the Role of the Chromosome 9q21.32 Locus in the Susceptibility of Diabetic Nephropathy
Source: PLoS One. 2013 Mar 29;8(3):e60301. doi: 10.1371/journal.pone.0060301 (PMC3612041; doi:10.1371/journal.pone.0060301)
Supplement: Table S9 — Single marker family-based association analyses between haplotype tagging SNPs across the four GoKinD loci and logACR among all family members. Affecteds and unaffecteds analyses are presented. (DOC) [file pone.0060301.s009.doc]

**Table S9.** Single marker family-based association analyses between haplotype tagging SNPs across the four GoKinD loci and logACR among all family members. Affecteds and unaffecteds analyses are presented.

| SNP | Chr. | Allele | Allele Frequency | # Families | S-E(S) | Var(S) | Z score | *P*-value  (adjusted *P*-value) |
| --- | --- | --- | --- | --- | --- | --- | --- | --- |
| rs39077 | 7p14.3 | A | 0.616 | 54 | -21.82 | 719.21 | -0.81 | 0.416 |
|  |  | C | 0.384 | 54 | 21.82 | 719.21 | 0.81 | (1.00) |
| rs17679605 | 7p14.3 | T | 0.835 | 41 | -4.14 | 398.09 | -0.21 | 0.836 |
|  |  | C | 0.165 | 41 | 4.14 | 398.09 | 0.21 | (1.00) |
| rs1929547 | 9q21.32 | T | 0.825 | 46 | 13.01 | 622.81 | 0.52 | 0.602 |
|  |  | G | 0.175 | 46 | -13.01 | 622.81 | -0.52 | (1.00) |
| rs12793371 | 11p15.4 | A | 0.676 | 56 | 18.44 | 522.10 | 0.81 | 0.420 |
|  |  | G | 0.324 | 56 | -18.44 | 522.10 | -0.81 | (1.00) |
| rs417957 | 11p15.4 | A | 0.553 | 56 | -6.36 | 551.56 | -0.27 | 0.787 |
|  |  | G | 0.447 | 56 | 6.36 | 551.56 | 0.27 | (1.00) |
| rs9555618 | 13q33.3 | G | 0.565 | 58 | -16.30 | 932.23 | -0.53 | 0.593 |
|  |  | A | 0.435 | 58 | 16.30 | 932.23 | 0.53 | (1.00) |
| rs7989975 | 13q33.3 | A | 0.837 | 36 | -6.56 | 344.95 | -0.35 | 0.724 |
|  |  | C | 0.163 | 36 | 6.56 | 344.95 | 0.35 | (1.00) |

# Families = number of nuclear families informative for the FBAT analysis

S-E(S) = observed minus the expected transmission for each allele

Var(S) = variance of the observed transmission for each allele

Z score: positive values indicate risk alleles, negative values indicate protective alleles
